# Using Green Solvents for Phase Inversion of PVDF/TiO_2_ Hybrid Coatings for Gas Phase Photocatalysis

**DOI:** 10.3390/molecules30081700

**Published:** 2025-04-10

**Authors:** Ewoud Cosaert, Hadis Mortazavi Milani, Geraldine J. Heynderickx, Dirk Poelman

**Affiliations:** 1 LumiLab, Department of Solid State Sciences, Ghent University, 9000 Ghent, Belgium; ewoud.cosaert@ugent.be (E.C.); seyedehhadis.mortazavimilani@ugent.be (H.M.M.); 2 Laboratory for Chemical Technology, Ghent University, 9000 Ghent, Belgium; geraldine.heynderickx@ugent.be

**Keywords:** photocatalysis, PVDF, TiO2, NMP, TEP, Rhodiasolv^®^ PolarClean, PC, phase inversion

## Abstract

Long-time exposure to volatile organic compounds (VOCs) in the atmosphere can have negative health effects on humans and other living organisms. In order to purify ambient air, these VOCs can be degraded using photocatalysis. In this research, commercially available TiO_2_ nanoparticles were immobilized in a porous poly(vinylidene fluoride-co-hexa-fluoropropylene) (PVDF) polymer matrix, synthesized using the phase inversion method. The most used solvent for PVDF is N-methyl-2-pyrrolidone (NMP). However, this solvent is known to be harmful to humans and the environment, and there is a need to replace NMP with a more ecological ‘green’ solvent. Here, triethyl phosphate (TEP), methyl-5-(dimethylamino)-2-methyl-5-oxopentanoate (Rhodiasolv^®^ PolarClean) and propylene carbonate (PC) were used to dissolve PVDF for the phase inversion synthesis of porous photocatalytically active PVDF/TiO_2_ hybrid layers onto aluminium slides. The photocatalytic degradation under UV (365 nm) of gaseous ethanol in an argon/oxygen (Ar/O_2_) atmosphere shows that these solvents are suitable replacements for NMP, but optimization is required to improve the performance of the layers. Apart from changing the solvent for PVDF, the UV and photocatalysis stability of PVDF has been determined, as well as the repeatability of the photocatalytic reaction, to prove that PVDF is a suitable polymer for this application.

## 1. Introduction

Volatile organic compounds (VOCs) in the air form a health issue for humans upon long exposure, such as an increased risk of cancer [1] and pulmonary diseases [2]. Risks are not only limited to gaseous VOCs, but polluted water can also induce health issues, including liver, kidneys, spleen and stomach problems [3], and environmental problems, such as negative effects on plant growth [4].

In the last few decades, the principle of photocatalysis [5] has proven its use in different fields, not only for pollutant degradation in air [6] or water [7], but also for H_2_ production [8], degrading microplastics [9] and inactivation of microorganisms [10], with TiO_2_ being one of the most used photocatalysts [11,12]. Currently, several companies provide commercial TiO_2_ photocatalysts in powder form, including Evonik Industries, KRONOS and Tronox. It remains challenging, however, to immobilize these (nano)powders to prepare efficient photocatalytic layers that can be used in different applications, in order to prevent the photocatalyst from being washed or blown away during photocatalytic operation.

The phase inversion or phase separation method has been widely investigated to synthesize porous flexible (PVDF) membranes for filtration applications [13]. This method has also been combined with a photocatalyst for synthesizing photocatalytically active flexible membranes used for water purification [14]. Only limited research has been performed using this method to prepare porous polymer samples embedded with a photocatalyst for degradation in the gas phase. Hwang et al. [15] and Magnone et al. [16] used the phase inversion method to prepare α-Al_2_O_3_ hollow fiber membranes. In their work, TiO_2_ was deposited on these membranes by a dip-coating method to study the photocatalytic degradation of gaseous ammonia. Dissolving polymers such as PVDF requires suitable solvents. The most conventional solvent for PVDF is NMP. This solvent, however, poses significant risks to human health and pollutes the environment when released in the atmosphere or in wastewater [17]. The need for less harmful and more ecologically friendly solvents is a key issue to decrease health and environmental issues and to allow large-scale application of the technology.

In the present research, layers prepared using the phase inversion method were combined with a commercially available nanopowder photocatalyst to synthesize fixed durable porous polymer (PVDF)/TiO_2_ hybrid layers on aluminium substrates. Different green solvents were used for this synthesis, as well as the more conventional, but hazardous, NMP. Photocatalytic performance was tested for gaseous ethanol in an Ar/O_2_ atmosphere. Additionally, the UV stability and the photocatalysis stability of PVDF were investigated. For the latter, the repeatability of the photocatalytic reaction was investigated to test the influence of photocatalysis by TiO_2_ on the polymer structure.

## 2. Results and Discussion

### 2.1. Structural Analysis

**Figure 1:**
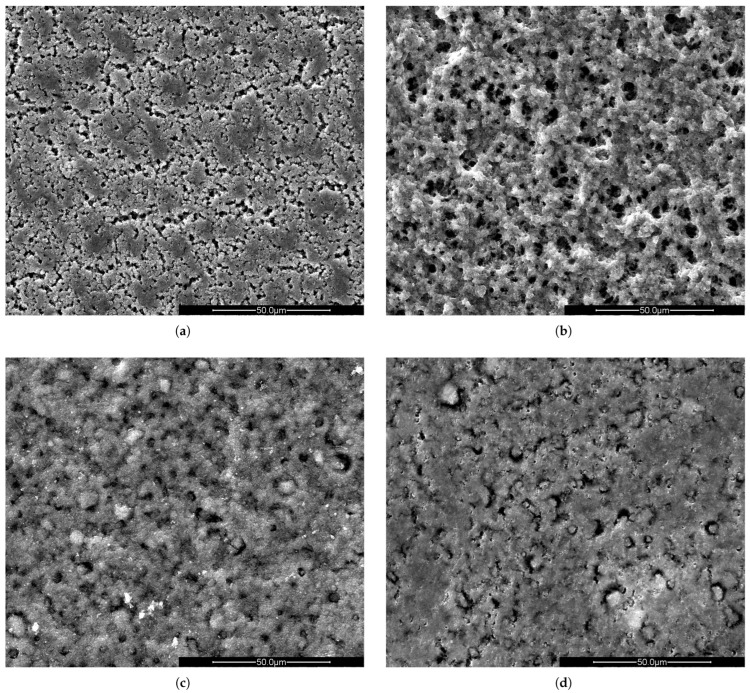
SEM images of PVDF/TiO_2_ hybrid layers on aluminium substrate synthesized using (**a**) PC, (**b**) Rhodiasolv^®^, (**c**) NMP and (**d**) TEP as solvent. The scale bar is 50 µm in all cases.

In Figure 1, SEM images of the PVDF/TiO_2_ layers, synthesized using four different solvents, are shown. Samples synthesized using Rhodiasolv^®^ (Figure 1b) and NMP (Figure 1c) show similar surface morphology and pore density, different from the samples synthesized using solvents PC (Figure 1a) and TEP (Figure 1d). This clearly indicates that changing the solvent influences the morphology of the PVDF/TiO_2_ layer. Most probably, different solvents have different exchange properties with DI water, resulting in a different layer structure.

In Table 1, the ratios between the Ti and F concentrations, measured with SEM-EDX (acceleration voltage 7 kV and 1.5 kV) and XPS, are shown for PVDF/TiO_2_ layers synthesized with different solvents. The ratio between Ti and F is expected to be 0.40, calculated from the amounts of TiO_2_ and PVDF used during synthesis. Comparing the ratios between the calculated atomic percentages of elements Ti (L-line) and F (K-line) from EDX (7 kV), a similar result was found for the four PVDF/TiO_2_ layers, slightly lower than the expected 0.40. This indicates that, as expected, the ratio between TiO_2_ and PVDF is similar for the different samples. When the acceleration voltage of the electrons in the scanning electron microscope is 7 kV, it can be simulated (using CASINO v2.48 [18]) that the information depth of EDX in these PVDF/TiO_2_ layers is up to 500 nm. By lowering the acceleration voltage of the electrons to 1.5 kV, the information depth decreases to about 40 nm. This gives lower values for the ratio between the Ti (L-line) and F (K-line) concentration, but again, no significant changes are observed between the different layers, with the layer synthesized with NMP showing a slightly higher ratio. This would indicate that only a limited amount of TiO_2_ is available for reaction at the polymer surface. Since for the photocatalytic degradation, only TiO_2_ located at the surface of the polymer matrix contributes, XPS was performed to quantize the TiO_2_ concentration at the surface from the Ti (2p) and F (1s) lines. This technique has an information depth of 1–5 nm. Comparing the different techniques, it is clear that the more surface-sensitive the technique becomes, the lower the ratio between Ti and F concentrations becomes. This means that there is less TiO_2_ at the surface of the layers. Also, a significant difference can be seen between the different layers.

### 2.2. Diffuse Reflectance

**Figure 2:**
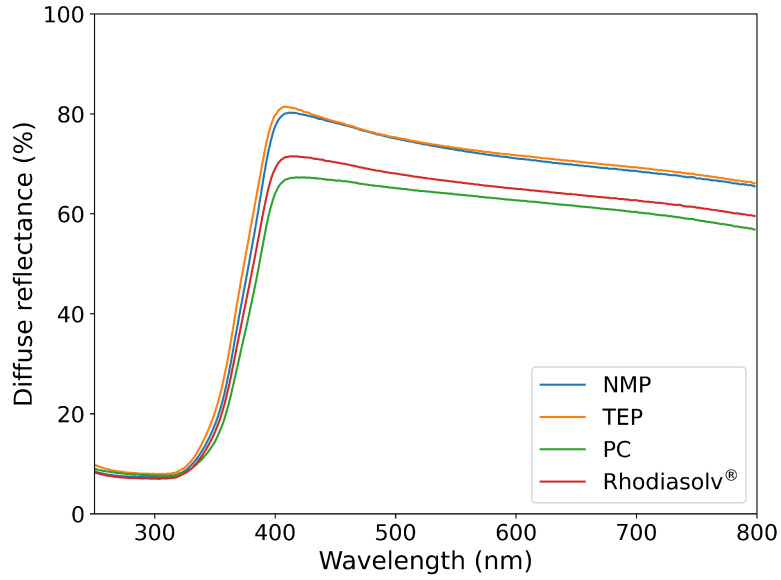
Diffuse reflectance of the PVDF/TiO_2_ hybrid layers, synthesized using four different solvents.

In Figure 2, the diffuse reflectance spectra are shown for the PVDF/TiO_2_ layers, synthesized using different solvents. All samples show strong absorption of UV light (365 nm) due to the presence of TiO_2_ nanoparticles [19], which allows these samples to be photocatalytically active. The absolute differences in reflection between different samples are most probably due to slight differences in thickness of the layers.

### 2.3. Photocatalytic Degradation of Ethanol

**Figure 3:**
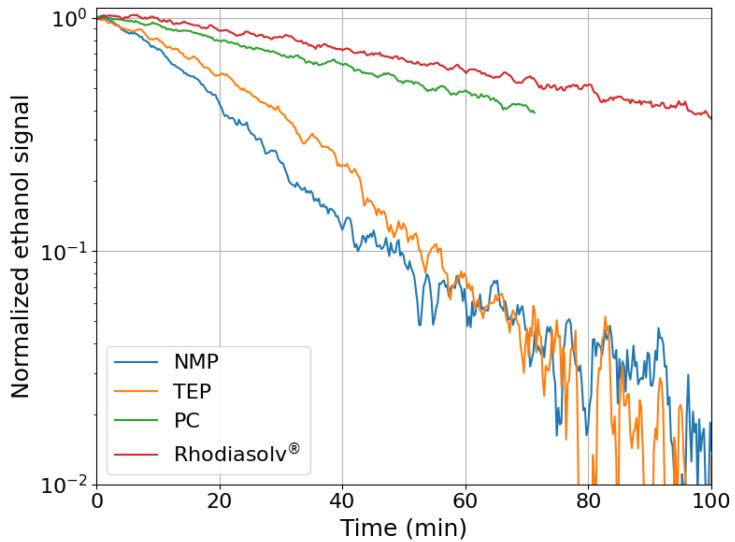
Photocatalytic degradation of ethanol of PVDF/TiO_2_ hybrid layers, synthesized using four different solvents.

In Figure 3, the photocatalytic decomposition of ethanol is shown for samples synthesized using different solvents. The layers prepared by using NMP and TEP as solvents show a similar photocatalytic degradation of ethanol. Using the samples prepared with Rhodiasolv^®^ and PC results in a much lower decomposition rate. From this, it can be concluded that substituting NMP with a green solvent can result in a layer that is photocatalytically active. However, decomposition rates differ significantly, so optimization of the synthesis method is necessary to also increase the decomposition rate for the Rhodiasolv^®^ and PC synthesized samples. The differences could be attributed to a different solubility of PVDF in the different solvents. Both PC and Rhodiasolv^®^ solutions had to be heated to 100 °C to completely dissolve PVDF. Because the casting and immersion process is still performed at room temperature, the viscosity of these cast solutions is much higher compared to the NMP and TEP solutions.

The ethanol degradation results cannot be related to the ratios between the Ti and F concentrations in Table 1. It is expected that a higher ratio, i.e., a higher concentration of TiO_2_ at the surface, would result in a higher ethanol decomposition rate. This expectation is not confirmed by the presented results. Possibly, the differences in layer porosity (allowing gas penetration into the layer or not) or surface defects (resulting in differences in electron/hole recombination rate) are of more importance for the photocatalytic activity than the actual TiO_2_ concentration at the top surface of the layers.

### 2.4. PVDF UV and Photocatalysis Stability

**Figure 4:**
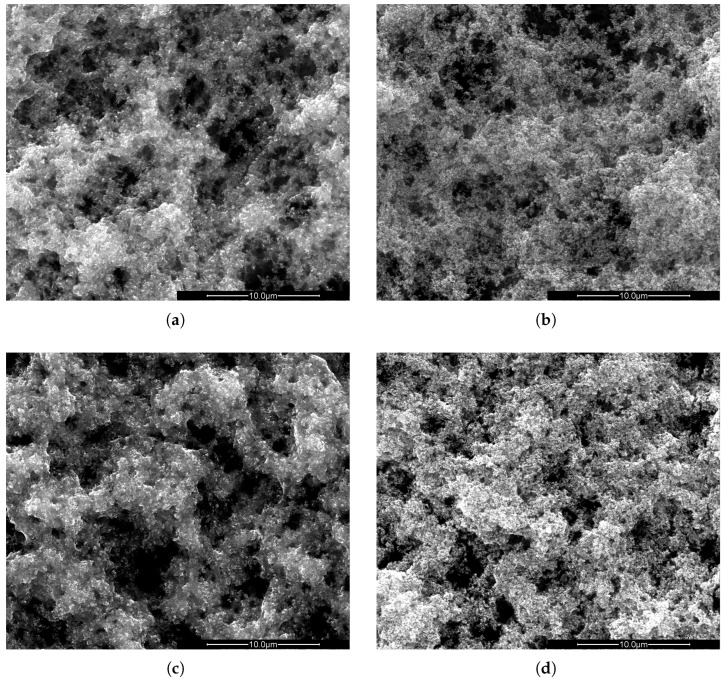
SEM images of PVDF/TiO_2_ hybrid layers on aluminium substrate synthesized using NMP (**a**) before and (**b**) after UV irradiation and synthesized using Rhodiasolv^®^ (**c**) before and (**d**) after UV irradiation. The scale bar is 10 µm in all cases.

PVDF polymer was chosen because it is known to be UV resistant and stable in different weather conditions with variable relative humidity and temperature [20,21,22]. In the present research, the UV resistance was also investigated in the presence of TiO_2_ in the layer. These experiments were conducted to check if the photocatalytic reaction itself could lead to degradation of PVDF, an effect that was previously observed in polylactic acid (PLA) [23]. The SEM images of PVDF/TiO_2_ samples are shown in Figure 4. Figure 4a,b are the layers prepared with NMP without and after UV irradiation, respectively. Similar images for the layers prepared with Rhodiasolv^®^ are shown in Figure 4c,d, without and after UV irradiation, respectively. According to these images, after 21 h of irradiation with a high-power UV LED (365 nm), the structure of the PVDF/TiO_2_ layer on the aluminium substrate did not change. This property, obviously, is very important for the application of the layers, since long-time UV irradiation should not influence the structure and composition of the PVDF/TiO_2_ layers. As mentioned in Section 3.3, a UV light source with a power density of 140 times the power density of the UV part of the solar spectrum (280 nm–380 nm) is used. Since this radiation does not degrade the PVDF/TiO_2_ layers, it can be concluded that these layers will also be stable upon irradiation with UV light from the sun.

**Figure 5:**
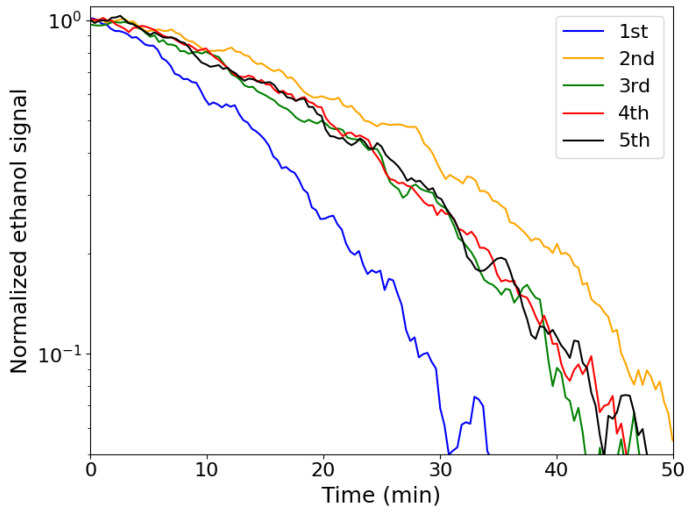
Repeatability measurements of the photocatalytic ethanol degradation using a PVDF/TiO_2_ hybrid layer onto aluminium substrate, synthesized using NMP.

Furthermore, the PVDF should also be resistant to the photocatalytic activity of the TiO_2_ photocatalytic nanoparticles. In Figure 5, the repeatability measurement of a PVDF/TiO_2_ layer onto aluminium substrate, synthesized using NMP, is shown. Photocatalytic ethanol degradation was performed five times consecutively using the same PVDF/TiO_2_ layer. It is clear that there is a slight difference between the first and second measurement, the second showing a slower degradation rate than the first. This could be related to residue solvent that did not evaporate from the layer. From the third photocatalytic measurement, the rate does not change, confirming that the PVDF/TiO_2_ layer does not deteriorate in the timescale of these measurements. In conclusion, a PVDF/TiO_2_ composite is suitable for efficiently degrading ethanol upon UV irradiation, maintaining its structure while doing so.

## 3. Materials and Methods

### 3.1. Materials

Poly(vinylidene fluoride-co-hexa-fluoropropylene) (PVDF, average M*_W_* = 400,000 g/mol, Sigma-Aldrich, St. Louis, MO, USA) was used as a polymer matrix in which titanium dioxide (TiO_2_ P25, Evonik Industries, Essen, Germany) was immobilized as a photocatalyst. For the phase inversion method, four different solvents were tested: N-methyl-2-pyrrolidone (NMP, ≥99%, Sigma-Aldrich), triethyl phosphate (TEP, ≥99.8%, Sigma-Aldrich), methyl-5-(dimethylamino)-2-methyl-5-oxopentanoate (Rhodiasolv^®^ PolarClean, Solvay, Brussels, Belgium) and propylene carbonate (PC, anhydrous 99.7%, Sigma-Aldrich). The last three materials are known as green solvents, since their toxicity and ecological impact are much lower than those of solvents such as NMP. Deionized (DI) water was used as a non-solvent for the phase inversion method. Pure ethanol (100%, Chem-Lab, Zedelgem, Belgium) was used as VOC for the photocatalytic degradation experiments. These materials were used as received, without any further purification.

### 3.2. Synthesis

TiO_2_ nanoparticles were immobilized in a porous PVDF matrix, using the phase inversion method. First, an amount of TiO_2_ was added to PVDF pellets in a mass ratio of 1:1. The solvent was added to this mixture in a mass ratio of PVDF to solvent of 1:9. After overnight mixing using a magnetic stirrer at 200 rpm, the polymer was fully dissolved, and the samples could be prepared. The NMP and TEP solutions were stirred at room temperature. The PC and Rhodiasolv^®^ solutions needed to be heated to 100 °C during stirring for a few hours for complete dissolution. This elevated temperature does not have an influence on the molecular structure of the formed PVDF/TiO_2_ hybrid layers, since it is well below the melting temperature (171–180 °C) and crystallization temperature (141–151 °C) of PVDF [24].

Using a casting knife, the before-mentioned solution was spread as a 100 μm thick layer onto an aluminium slide (dimensions (L × W × H): 75 mm × 25 mm × 1 mm). After deposition of this solution, the aluminium slide was immersed for 15 s in 250 mL DI water to apply the phase inversion method. To finalize the synthesis, all samples were dried overnight under atmospheric conditions in a fume hood, so any leftover solvent or DI water in the layers could evaporate.

### 3.3. Experimental Setup

Scanning electron microscopy (SEM) was performed to obtain information on the surface structure and morphology of the PVDF layers containing photocatalytic TiO_2_ powder, using a FEI (Hillsboro, OR, USA)Quanta 200 F instrument at high vacuum and with an acceleration voltage of 20 kV. Energy dispersive X-ray spectroscopy (EDX) was performed using a Jeol (Akishima, Tokyo, Japan) JSM-IT800 SEM with an Oxford Instruments (Abingdon, Oxfordshire, UK) Xplore 30 detector to quantify the concentration of the TiO_2_ powder in the polymer matrix, using AZtec software 6.2. The ratio between the titanium (Ti) and fluorine (F) concentrations was determined as an average of two measurements. For Ti, the L-line was used because the concentration was also determined using an electron acceleration voltage of 1.5 kV. For F, the K-line was used.

X-ray photoelectron spectroscopy (XPS) was performed on a Thermo Fisher Scientific™ (Waltham, MA, USA) Sigma Probe instrument (10^−10^ mbar base pressure) using monochromatic Al Kα (1486.6 eV) radiation generated at 15 kV, focused into a 300 μm spot onto the sample surface. Survey scans were acquired at 200 eV pass energy with a 0.5 eV step size, from which the concentration of Ti and F were determined with the CasaXPS software package v2.3.26 [25], using the Ti (2p) and the F (1s) lines, respectively. Charging of the sample required neutralization, which was ensured by operating a Thermo Fisher FG02 dual beam (using both electrons and argon ions) flood gun at an electron energy of 1.4 V and a current of 50 μA.

Diffuse reflectance spectra were measured using a Perkin Elmer (Waltham, MA, USA) Lambda 1050 UV-Vis-NIR spectrophotometer with an integrating sphere to obtain absorption properties of the synthesized samples.

The photocatalytic degradation of ethanol was measured in a stainless steel batch reactor, previously described in more detail [19]. This reactor consists of a pumping system (rotary and turbomolecular pumps—Pfeiffer Vacuum, Aßlar, Germany), a sample heating stage, a high-power UV LED with a collimating mirror (365 nm, 3.8 W/13 W optical/electrical power, CUN6HF4A—Seoul Viosys, Ansan-si, Gyeonggi-do, Republic of Korea) illuminating the photocatalyst through a quartz window and a quadrupole mass spectrometer (QMS) (Hiden Analytical ExQ, Warrington, UK) to analyze the gas composition in the reactor. First, after mounting the sample, the reactor chamber is evacuated and the gas lines are flushed with Ar. Then, the chamber is disconnected from the pumping system, obtaining a batch reactor. Using mass flow controllers (Bronkhorst, Ruurlo, The Netherlands), Ar/O_2_ (80 vol%/20 vol%) was added to a slight overpressure of 1050 mbar to avoid air leaking in. After reaching an adsorption/desorption balance of Ar/O_2_ in the reactor/photocatalyst and obtaining a temperature of the sample holder of 40 °C to avoid temperature fluctuations during measurements, 6 μL of ethanol was injected, corresponding to a concentration of 273 ppm, as described in more detail in [26]. During a stabilization process of about 40 min, it was clear that the ethanol signal remained constant, which means that ethanol was not decomposed in the dark, without UV irradiation. After this stabilization of the gas mixture, the measurements were initiated by turning on the high-power UV LED and measuring different mass-to-charge ratio (*m*/*z*) signals corresponding to ethanol, carbon dioxide (CO_2_), oxygen (O_2_), argon (Ar) and water, as a function of time. From the decrease in the ethanol signal, the photocatalytic performance of each layer was evaluated. The ethanol signal was measured at a mass-to-charge ratio of 45 amu, the CO_2_ signal at 44 amu. However, ethanol also has a small contribution to the 44 amu signal, while CO_2_ also has a small contribution to the 45 amu signal. So, for ethanol decomposition analysis, it is important to take the CO_2_ contribution to the 45 amu signal into account and calculate the actual signal of ethanol. First, the ratio between the 44 amu signal and the 45 amu signal of ethanol is acquired from the mass spectrum of ethanol from the NIST database [27]. Likewise, the ratio between the 45 amu signal and the 44 amu signal of CO_2_ is acquired from the mass spectrum of CO_2_ from the NIST database [28]. With this information, the actual signal of ethanol can be calculated using the signals measured with the QMS.

To investigate the UV stability and photocatalysis stability of the photocatalytic layers, two PVDF/TiO_2_ layers on aluminium substrates were prepared using the synthesis method described above. For one sample, NMP was used to dissolve PVDF, and for the other, Rhodiasolv^®^. After synthesis of the layers, the aluminium substrates were cut in two pieces. One piece was illuminated by UV light (365 nm) for 21 h in an Ar/O_2_ (80 vol%/20 vol%) atmosphere, the other piece was used as a reference. This type of measurement can be used to investigate how the PVDF/TiO_2_ structure changes after UV illumination. This includes the UV stability of the PVDF but also the stability of PVDF in combination with TiO_2_.

The optical power density of the UV LED, combined with a collimating mirror, used in this research, was compared to the solar irradiance. The optical power density of the UV LED with mirror that irradiates the PVDF/TiO_2_ layer was measured to be about 2800 W/m^2^ when using a thermal power sensor (Thorlabs S401C, Newton, NJ, USA). The part of the solar spectrum that can be used to activate TiO_2_ ranges approximately from 280 nm to 380 nm. The total solar power density in this wavelength region is about 20 W/m^2^ [29]. The power density of the UV LED (365 nm) is about 140 times that of the 280 nm–380 nm part of the solar spectrum. Thus, the UV LED, used both for the photocatalytic degradation of ethanol and the stability tests of PVDF, has a UV power density equivalent to 140 suns.

Additionally, the repeatability of the photocatalytic reaction of a porous PVDF/TiO_2_ layer on an aluminium substrate synthesized using NMP was tested five times consecutively, similarly to the ethanol degradation experiments explained above. In between two consecutive measurements, the reactor was evacuated and Ar/O_2_ was introduced, followed by ethanol injection. This repeatability is performed, on the one hand, to confirm that these layers do not lose photocatalytic activity, and on the other hand, to make sure the PVDF structure is not deteriorated by UV or by photocatalytic activity from TiO_2_. For the latter, if the PVDF structure would deteriorate, more TiO_2_ embedded in the structure would become exposed to the surrounding atmosphere, resulting in a faster ethanol degradation rate during photocatalytic measurements [23]. If the photocatalytic degradation rate of ethanol is not significantly changed after repeatedly using the same PVDF/TiO_2_ layer, it can be concluded that no significant degradation of the PVDF occurs, either by exposure to UV light or as a result of the photocatalytic activity of the TiO_2_ nanoparticles.

## 4. Conclusions

It was proved that PVDF/TiO_2_ layers on aluminium substrates synthesized by the phase inversion method are suitable for gaseous ethanol degradation through photocatalytic reactions. Solvents that are less harmful than NMP for both human health and environment, namely TEP, Rhodiasolv^®^ PolarClean and PC, can be used to synthesize PVDF/TiO_2_ samples for ethanol degradation upon UV irradiation (365 nm). From the photocatalytic degradation experiments, TEP was found to be the best competitor for NMP. However, the performance of the samples prepared with PC and Rhodiasolv^®^ still lags behind the conventional NMP sample. More research, such as varying solvent concentration and solution viscosities, is necessary for the optimization of the synthesis method, such that synthesis using the green solvent becomes an even better competitor for the conventional NMP.

Apart from the photocatalytic decomposition, the composition of these layers was investigated using SEM-EDX, confirming a porous polymer matrix and obtaining ratios between the Ti and F concentrations in these layers. XPS measurements resulted in ratios between the Ti and F concentrations at the surface of the layers. These were significantly smaller than results obtained by using SEM-EDX, proving that the ratio between TiO_2_ and PVDF is lower at the surface.

Furthermore, the UV stability and photocatalysis stability of PVDF experiments showed that PVDF is suitable to be combined with UV light and a photocatalyst, such as TiO_2_. The polymer is not prone to degradation, either due to the UV light directly or from the photocatalytic reaction from the TiO_2_, in the timescale of the degradation measurements performed in this research. This last property was proved by repeatedly performing photocatalytic degradation measurements and comparing the ethanol degradation.

## Figures and Tables

**Table 1:** Ratios between the Ti and F concentrations obtained using SEM-EDX with two different electron acceleration voltages and using XPS for the PVDF/TiO_2_ layers synthesized with a different solvent.

	NMP	TEP	Rhodiasolv^®^	PC
EDX (7 kV)	0.30	0.31	0.32	0.27
EDX (1.5 kV)	0.25	0.20	0.20	0.21
XPS	0.06	0.03	0.04	0.10

## Data Availability

The data presented in this study are available on request from the corresponding author.

## Abbreviations

The following abbreviations are used in this manuscript:

DI	Deionized
EDX	Energy dispersive X-ray spectroscopy
LED	Light emitting diode
NMP	N-methyl-2-pyrrolidone
PC	Propylene carbonate
PLA	Polylactic acid
PVDF	Poly(vinylidene fluoride-co-hexa-fluoropropylene)
QMS	Quadrupole mass spectrometer
SEM	Scanning electron microscopy
TEP	Triethyl phosphate
UV	Ultraviolet
VOC	Volatile organic compound
XPS	X-ray photoelectron spectroscopy
